# In vivo near-infrared imaging of ErbB2 expressing breast tumors with dual-axes confocal endomicroscopy using a targeted peptide

**DOI:** 10.1038/s41598-017-13735-z

**Published:** 2017-10-31

**Authors:** Zhenghong Gao, Gaoming Li, Xue Li, Juan Zhou, Xiyu Duan, Jing Chen, Bishnu P. Joshi, Rork Kuick, Basma Khoury, Dafydd G. Thomas, Tina Fields, Michael S. Sabel, Henry D. Appelman, Quan Zhou, Haijun Li, Ken Kozloff, Thomas D. Wang

**Affiliations:** 1 Dept of Internal Medicine, University of Michigan, Ann Arbor, MI 48109 United States; 2 Dept of Biomedical Engineering, University of Michigan, Ann Arbor, MI 48109 United States; 3 Dept of Biostatistics, University of Michigan, Ann Arbor, MI 48109 United States; 4 Dept of Orthopaedic Surgery, University of Michigan, Ann Arbor, MI 48109 United States; 5 Dept of Pathology, University of Michigan, Ann Arbor, MI 48109 United States; 6 Dept of Surgery, University of Michigan, Ann Arbor, MI 48109 United States; 7 Dept of Mechanical Engineering, University of Michigan, Ann Arbor, MI 48109 United States

## Abstract

ErbB2 expression in early breast cancer can predict tumor aggressiveness and clinical outcomes in large patient populations. Accurate assessment with physical biopsy and conventional pathology can be limited by tumor heterogeneity. We aim to demonstrate real-time optical sectioning using a near-infrared labeled ErbB2 peptide that generates tumor-specific contrast in human xenograft breast tumors *in vivo*. We used IRDye800CW as the fluorophore, validated performance characteristics for specific peptide binding to cells *in vitro*, and investigated peak peptide uptake in tumors using photoacoustic tomography. We performed real-time optical imaging using a handheld dual-axes confocal fluorescence endomicroscope that collects light off-axis to reduce tissue scattering for greater imaging depths. Optical sections in either the vertical or horizontal plane were collected with sub-cellular resolution. Also, we found significantly greater peptide binding to pre-clinical xenograft breast cancer *in vivo* and to human specimens of invasive ductal carcinoma that express ErbB2 *ex vivo*. We used a scrambled peptide for control. Peptide biodistribution showed high tumor uptake by comparison with other organs to support safety. This novel integrated imaging strategy is promising for visualizing ErbB2 expression in breast tumors and serve as an adjunct during surgery to improve diagnostic accuracy, identify tumor margins, and stage early cancers.

## Introduction

Breast cancer is one of the most common carcinomas in the world with an estimated annual incidence and mortality of ~1.38 million and ~458,000, respectively, and is the most frequent cancer found in women^1^. Up to 30% of all breast cancers express ErbB2, also known as human epidermal growth factor receptor 2 (HER2)^2^. Increased ErbB2 expression has clinical significance for predicting more aggressive disease, greater likelihood of lymph node involvement, and poor clinical outcomes^2,3^. Receptor status can be used to guide choice of therapy and to monitor treatment effectiveness^3^. Immunohistochemistry is commonly used to evaluate ErbB2 expression in either surgical or biopsy specimens. However, tumor heterogeneity found either within the same lesion or among different lesions in the same patient may limit the usefulness of this technique^4^. Furthermore, expression levels may differ between primary breast cancers and local lymph node metastases. While surgery is often used to remove macroscopic tumors, residual microscopic foci can lead to positive margins and need for re-excision^5^. These limitations highlight the need for a specific targeting ligand that can assess ErbB2 status and can be detected with sub-cellular resolution, deep tissue penetration, and real-time speed. Clinical applications include image-guided surgery, detection of cancer-positive lymph nodes, assessing tumor margins, and identifying new lesions.

Molecular imaging is an emerging methodology that uses exogenous imaging agents to detect target overexpression. Whole body methods, such as PET, SPECT, and MRI, are used most often^6^. Optical methods offer a number of advantages in resolution and speed for real time, intra-operative visualization of breast cancer, including tumor margins and lymph node metastases^7^. The dual-axes confocal design uses separate illumination and collection beams that travel along different light paths at an angle into the tissue, and the resulting region of overlap defines the focus^8^. This off-axis geometry minimizes the effects of tissue scattering, and enhances the dynamic range of detection so that images can be collected in either vertical or horizontal cross-sections^9,10^. The vertical orientation best displays the original tissue architecture, evaluates depth of tumor invasion, and is used by pathologists for early cancer staging^11^. Combined with a specific ligand, a handheld instrument can be used to rapidly assess target expression with depth, and is promising for clinic translation to support image-guided surgery.

Monoclonal antibodies, such as trastuzumab, have high affinity and specificity for ErbB2^12^. Commonly used clinically for breast cancer therapy, this antibody has recently been repurposed to visualize ErbB2 expression using PET and SPECT^13,14^. Antibodies are bulky in size, and have limited usefulness for diagnostic imaging because of slow binding kinetics, long circulatory half-life, and increased background^15^. Performance can also be impaired by slow tumor localization, heterogeneous distribution, and inadequate concentration^16^, while immunogenicity can limit repeated use. We have developed a peptide that binds specifically to ErbB2 that is labeled with IRDye800CW, a bright near-infrared (NIR) fluorophore, that maximizes tumor imaging depth^17^. We aim to use a handheld dual-axes confocal instrument to visualize peptide uptake in human xenograft breast tumors *in vivo*. We demonstrate feasibility for this integrated imaging methodology to be used as an adjunct in breast cancer surgery for real-time detection of ErbB2 positive cancer cells in lymph nodes and to rapidly assess tumor margins.

## Results

### Peptide Specific for ErbB2 (HER2)

**Figure 1:**
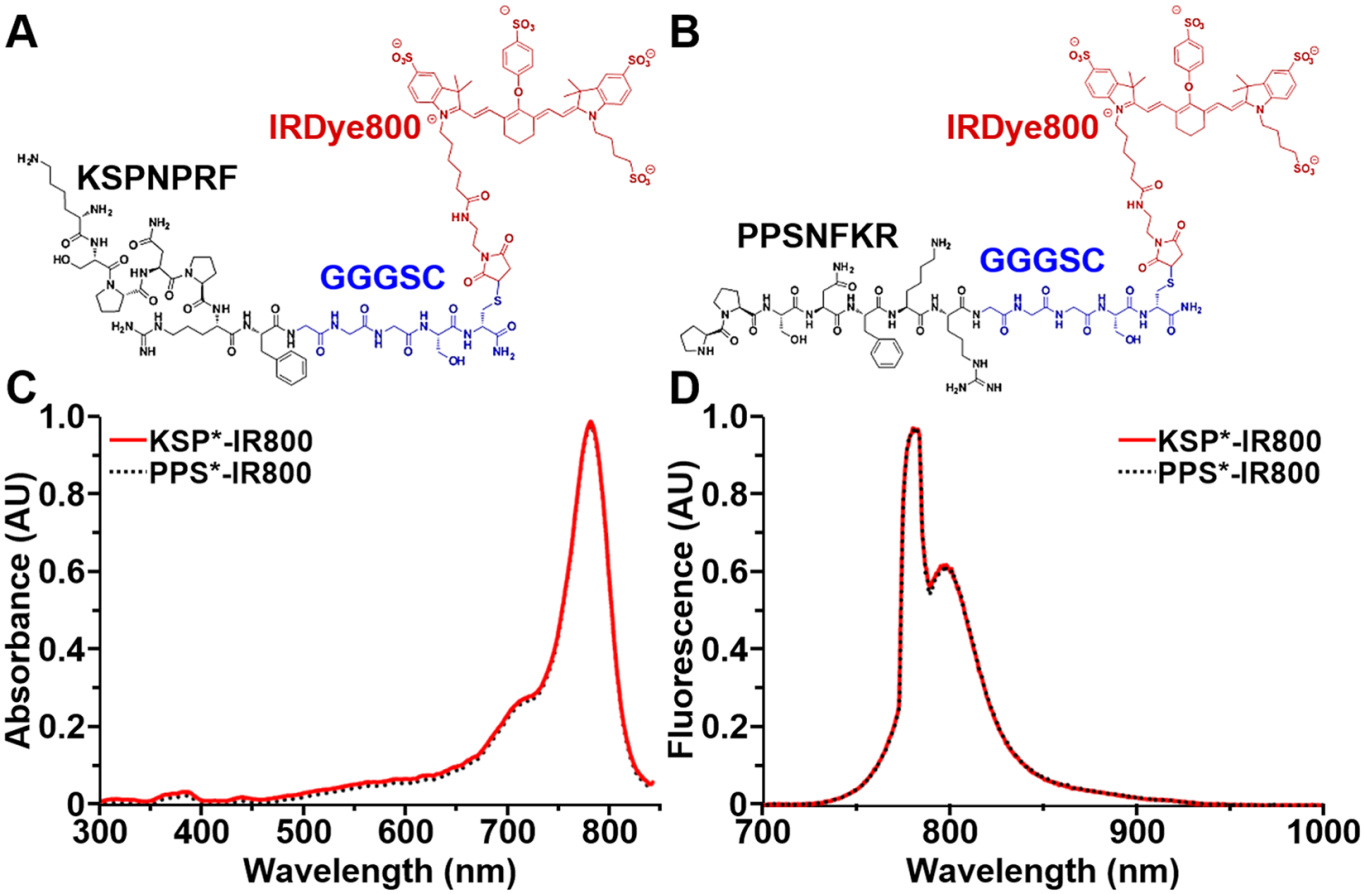
NIR-labeled peptides. (**A**) Biochemical structure of ErbB2-specific peptide KSPNPRF (black) labeled with IRDye800 (red) via a GGGSC linker (blue), hereafter KSP*-IR800. (**B**) Scrambled peptide PPSNFKR is used for control, hereafter PPS*-IR800. KSP*-IR800 and PPS*-IR800 have (**C**) maximum absorbance at λ_ex_ = 780 nm and (**D**) peak emission at λ_em_ = 800 nm.

We covalently linked the C-terminus of the linear, monomeric peptide KSPNPRF with IRDye800CW via a GGGSC linker, hereafter KSP*-IR800, Fig. 1A. The linker is used to increase structural flexibility, enhance binding interactions, and separate the peptide from the fluorophore to minimize steric hindrance. We similarly labeled the scrambled sequence PPSNFKR for control, hereafter PPS*-IR800, Fig. 1B. We achieved >95% purity by HPLC for both peptides. Using mass spectrometry, we measured a molecular weight of 2330.85 for KSP*-IR800 and PPS*-IR800 which agrees with the expected values and supports successful synthesis, Fig. S1A,B. The absorption spectra of both KSP*-IR800 and PPS*-IR800 have maximum absorbance at λ_ex_ = 780 nm, Fig. 1C, and peak emission at λ_em_ = 800 nm, Fig. 1D.

### Specific Peptide Binding to Cells Expressing ErbB2

**Figure 2:**
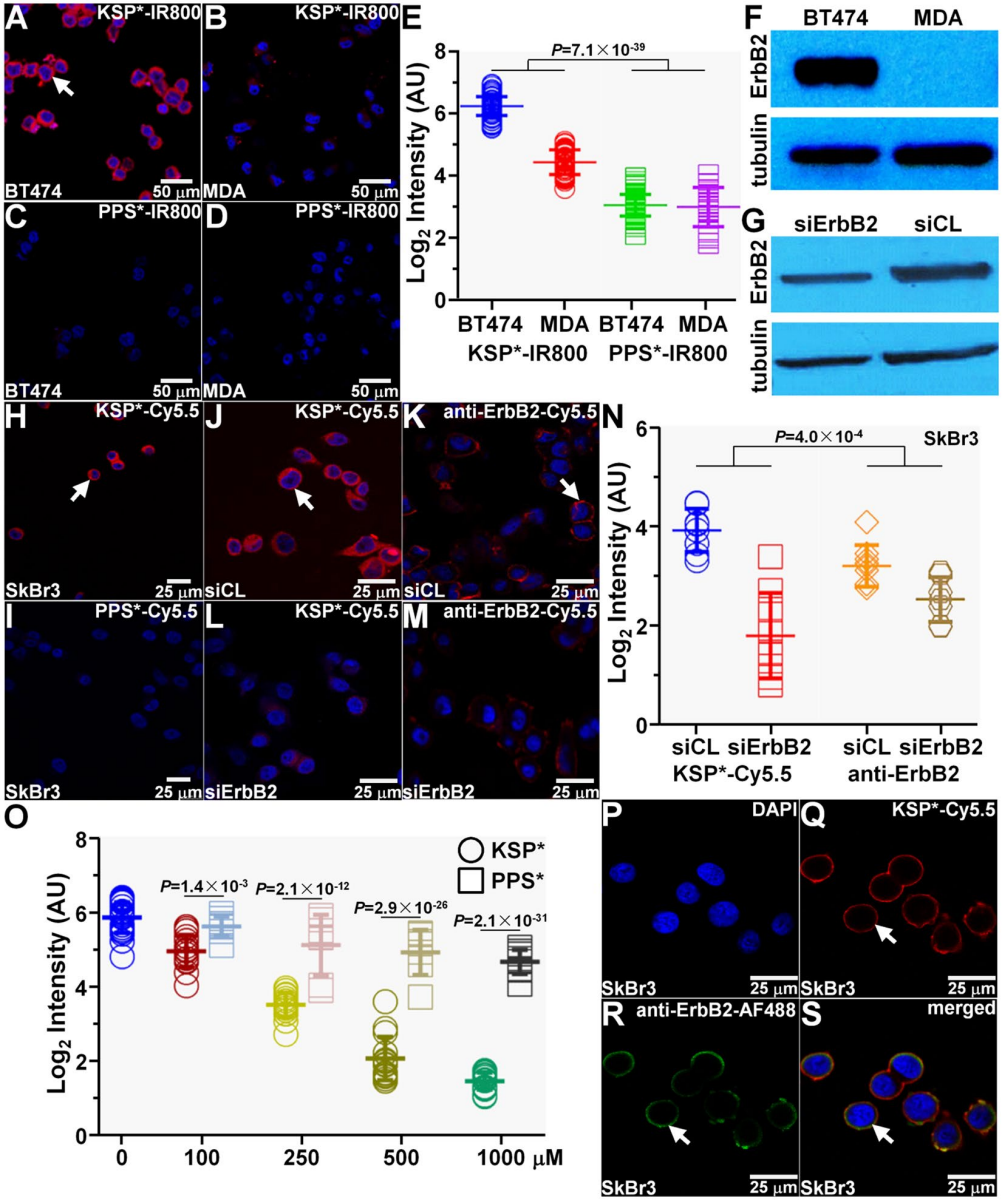
Specific peptide binding to ErbB2. KSP*-IR800 shows (**A**) strong staining to the surface (arrow) of BT474 human breast cancer cells but not to that for (**B**) MDA-MB-231. (**C**,**D**) Scrambled control peptide PPS*-IR800 shows minimal binding to either cell. (**E**) Quantitative comparison is shown in log_2_ scale. We found 3.55-fold greater signal for KSP*-IR800 with BT474 versus MDA-MB-231 cells but only a 1.04-fold difference with PPS*-IR800. Using an ANOVA model fit with terms for 4 means to log-transformed data, we found the difference of differences to be significant. Western blot analysis shows ErbB2 expression for (**F**) BT474 and MDA-MB-231 cell and for (**G**) SKBR3 human breast cancer cells transfected with siErbB2 targeting siRNA (knockdown) and siCL non-targeting siRNA (control). (**H**,**I**) Use of a Cy5.5 label produces results to that found for IRDye800 with SKBR3 cells. (**J**,**K**) KSP*-Cy5.5 and anti-ErbB2-Cy5.5 bind significantly greater to the surface (arrows) of siCL (control) SKBR3 cells compared with that for (**L**,**M**) siErbB2 (knockdown) cells. (**N**) We found 4.42-fold greater signal for KSP*-Cy5.5 with siCL treated SkBr3SKBR3 control cells compared with that for siErbB2 knockdown cells and 1.60-fold greater intensities with anti-ErbB2-Cy5.5. Using an ANOVA model, we fit with terms for 4 means to log-transformed data, and found the difference of differences to be significant. (**O**) On competition, we found the mean fluorescence intensity with KSP*-Cy5.5 to SKBR3 cells decreases significantly in a concentration dependent manner with addition of unlabeled KSP*. By comparison, binding was significantly less affected with addition of unlabeled PPS*. We used a one-way ANOVA to compare the mean intensities, and show *P*-values at each concentration. (**P**–**S**) Binding of KSP*-Cy5.5 (red) and anti-ErbB2-AF488 (green) co-localizes to the surface (arrows) of SKBR3 cells with a Pearson’s correlation coefficient of ρ = 0.70. Each result is an average of 3 independent measurements.

We used confocal microscopy to demonstrate peptide binding to the cell membrane. We observed strong signal for KSP*-IR800 to the surface (arrow) of BT474 (ErbB2+) but not MDA-MB-231 (ErbB2−) human breast cancer cells *in vitro*, Fig. 2A,B. Minimal signal was seen with PPS*-IR800 to either cell, Fig. 2C,D. We quantified the fluorescence intensities for all cells, and found a significantly greater result with KSP*-IR800 for BT474 versus MDA-MB-231 cells. This difference was significantly larger than that observed for the control peptide, Fig. 2E. Western blot analysis shows the ErbB2 expression level for each cell, Fig. 2F.

We performed siRNA knockdown of ErbB2 in SKBR3 human breast cancer cells to validate specific binding of KSP* to ErbB2. Western blot analysis shows ErbB2 expression is each cell line, Fig. 2G. We use Cy5.5 rather than IRDye800 to show that changing the fluorophore label does not affect peptide binding to the cell membrane, Fig. 2H,I. We observed strong binding of KSP*-Cy5.5 and Cy5.5-labeled anti-ErbB2 antibody to the surface (arrows) of control SKBR3 cells (siCL), Fig. 2J,K, but a significant reduction in fluorescence intensities for ErbB2 knockdown cells (siErbB2), Fig. 2L,M. Quantified results are shown, Fig. 2N.

We performed competition by adding either unlabeled KSP* or PPS* peptide to block binding of KSP*-IR800 to BT474 cells. We found the mean fluorescence intensities decreases significantly in a concentration dependent manner, Fig. 2O. Adding PPS* resulted in significantly less effective competition as compared with KSP*, Fig. 2O. These results demonstrate that binding occurs with the peptide and not the fluorophore. We also observed good co-localization of binding between KSP*-Cy5.5 (red) and AlexaFluor488-labeled anti-ErbB2 antibody (green) with Pearson’s correlation coefficient ρ = 0.70, Fig. 2P–S.

Using a structural model, labeling of KSP* with different organic fluorophores showed negligible effects on binding to ErbB2, Fig. S2A,B. We used KSP*-Cy5.5 for some *in vitro* studies because Cy5.5 is compatible with our core microscope facilities. Alternatively, we used KSP*-IR800 for all *in vivo* validation studies to achieve greater imaging depths.

### *In Vivo* Imaging of Xenograft Breast Tumors

**Figure 3:**
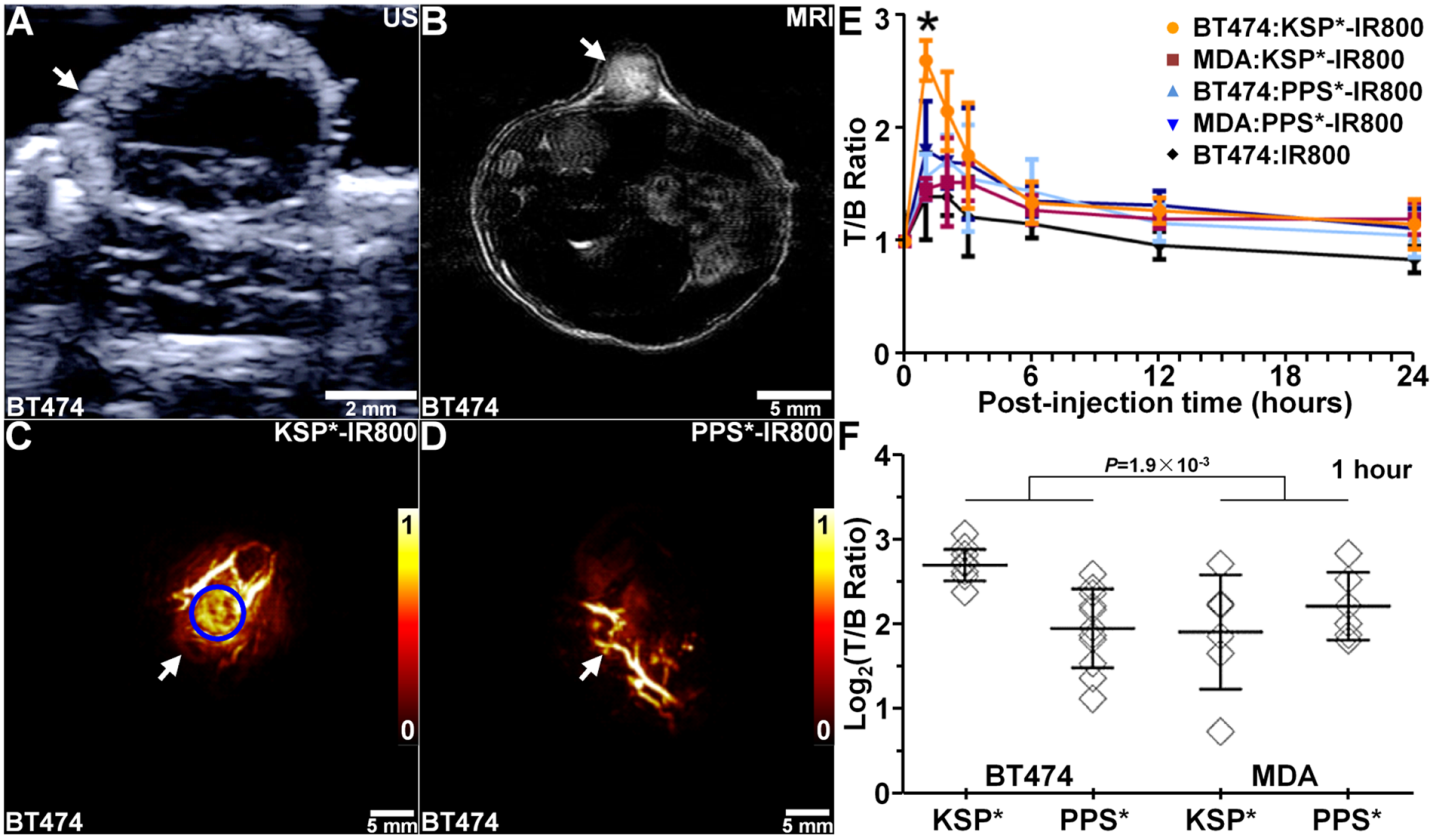
*In vivo* photoacoustic imaging. (**A**) Ultrasound (US) and (**B**) MR (T_1_-weighted, contrast-enhanced) images show structure of human xenograft breast (BT474) tumor (arrows) in nude mouse. Photoacoustic images collected at 1 hour post-injection of (**C**) KSP*-IR800 and (**D**) PPS*-IR800 show tumor expression of ErbB2 (arrows). (**E**) The T/B ratios from BT474 and MDA-MB-231 tumors over time show peak peptide uptake at 1 hour and return to baseline levels at ~24 hours post-injection. (**F**) T/B ratios from n = 11 tumors in n = 8 mice are shown at 1 hour post-injection. We fit an ANOVA model with terms for 4 means, and found 2.0-fold greater signal with KSP*-IR800 in BT474 versus MDA-MB-231 tumors. The difference of differences was significant, *P* = 1.9 × 10^−3^.

We developed human xenograft breast tumors with subcutaneous injection of BT474 and MDA-MB-231 cells in nude mice at 4 weeks of age. The BT474 tumors first appeared at ~4–5 weeks post-innoculation, and grew to a size >800 mm^3^ by 12 weeks. The MDA-MB-231 tumors reached a size >2000 mm^3^. We collected ultrasound (US), Fig. 3A, and MRI images, Fig. 3B, to measure the dimensions of BT474 tumors over time, Fig. S3A. For MDA-MB-231 tumors, we monitored tumor size using a micrometer, Fig. S3B. We imaged BT474 and MDA-MB-231 tumor bearing mice between 8–12 weeks and 2–8 weeks, respectively, after inoculation. Tumor dimensions varied between 0.2–1.2 × 10^3^ mm^3^ over these times.

We collected photoacoustic images to assess tumor uptake of peptide, Fig. S4A–F, and observed significantly greater signal for KSP*-IR800 versus PPS*-IR800, Fig. 3C,D. PPS*-IR800 was injected 2 days later in the same animal for control after the signal from KSP*-IR800 returned to baseline levels. The background is the same region of interest (ROI) as the tumor prior to injection of peptide, and is attributed to mainly to hemoglobin in the vasculature. We observed peak target-to-background (T/B) ratio at 1 hour post-injection of peptide with clearance (T/B ratio ∼1) by ∼24 hours, Fig. 3E. We also injected IRDye800 alone for control, and observed significantly lower signal with peak at 2 hours post-injection consistent with non-specific tumor uptake. We quantified the photoacoustic signal for each tumor, and found the mean T/B ratio with KSP*-IR800 to be significantly greater for BT474 versus MDA-MB-231 tumors. Also, this difference is greater than that observed for PPS*-IR800 at peak uptake (1 hour). Figure 3F.

**Figure 4:**
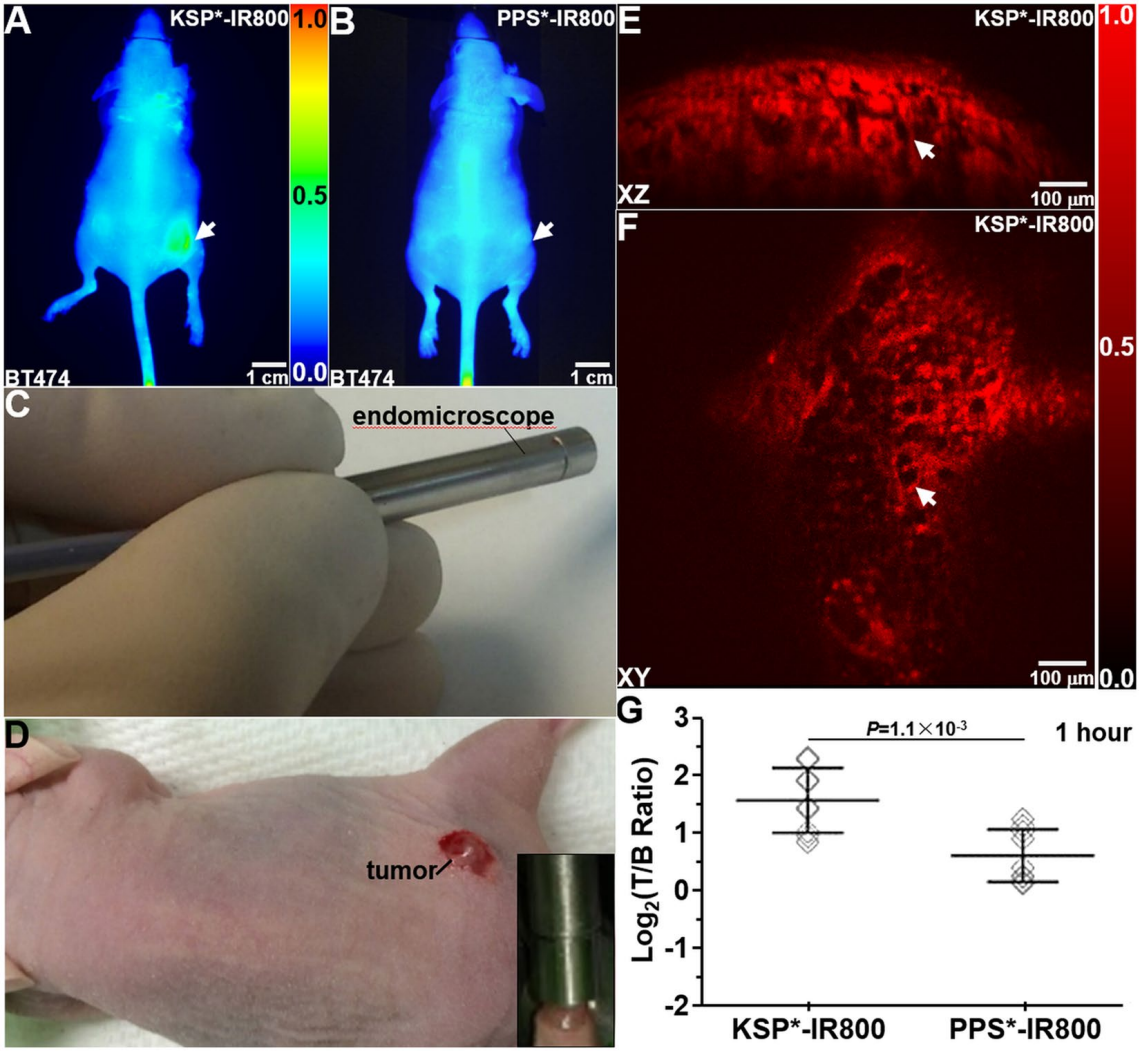
*In vivo* optical imaging. Whole body fluorescence images show increased uptake of (**A**) KSP*-IR800 compared with (**B**) PPS*-IR800 at 1 hour post-injection in human xenograft breast (BT474) tumor implanted in nude mouse. (**C**) Distal tip of dual-axes confocal endomicroscope was placed in contact (inset) with (**D**) tumor in live nude mouse. Optical sections collected in the (**E**) vertical (1000 × 430 μm^2^) and (**F**) horizontal (1000 × 1000 μm^2^) planes, respectively, show strong uptake of KSP*-IR800 in tumor (arrow). (**G**) At 1 hour post-injection, the mean T/B ratio for KSP*-IR800 was significantly greater than that for PPS*-IR800 in n = 9 tumors from n = 3 mice with a mean fold-difference of 2.0, *P* = 1.1 × 10^−3^ by paired, two-way t-test.

We collected whole body NIR fluorescence images at 1 hour after peptide injection to localize the tumor (arrows), Fig. 4A,B. We then placed the distal end of a dual-axes confocal endomicroscope, Fig. S5, in direct contact with the tumor, Fig. 4C,D (inset). We collected *in vivo* optical sections in either the vertical (XZ) or horizontal (XY) plane with a field-of-view (FOV) of either 1000 × 430 μm^2^ or 1000 × 1000 μm^2^, respectively, Fig. 4E,F. These NIR fluorescence images provided visualization of KSP*-IR800 uptake (arrows) in the tumor with sub-cellular resolution (vertical 5 μm or horizontal 2 μm). We quantified the fluorescence signal, and found a significantly higher T/B ratio for KSP*-IR800 versus PPS*-IR800 in n = 9 tumors from n = 3 mice, Fig. 4G. Real-time images in either the vertical (Visualization1) or horizontal (Visualization2) plane are used to present ErbB2 expression in a 3-dimensional (3D) volumetric image (Visualization3).

### ErbB2 Peptide Biodistribution

**Figure 5:**
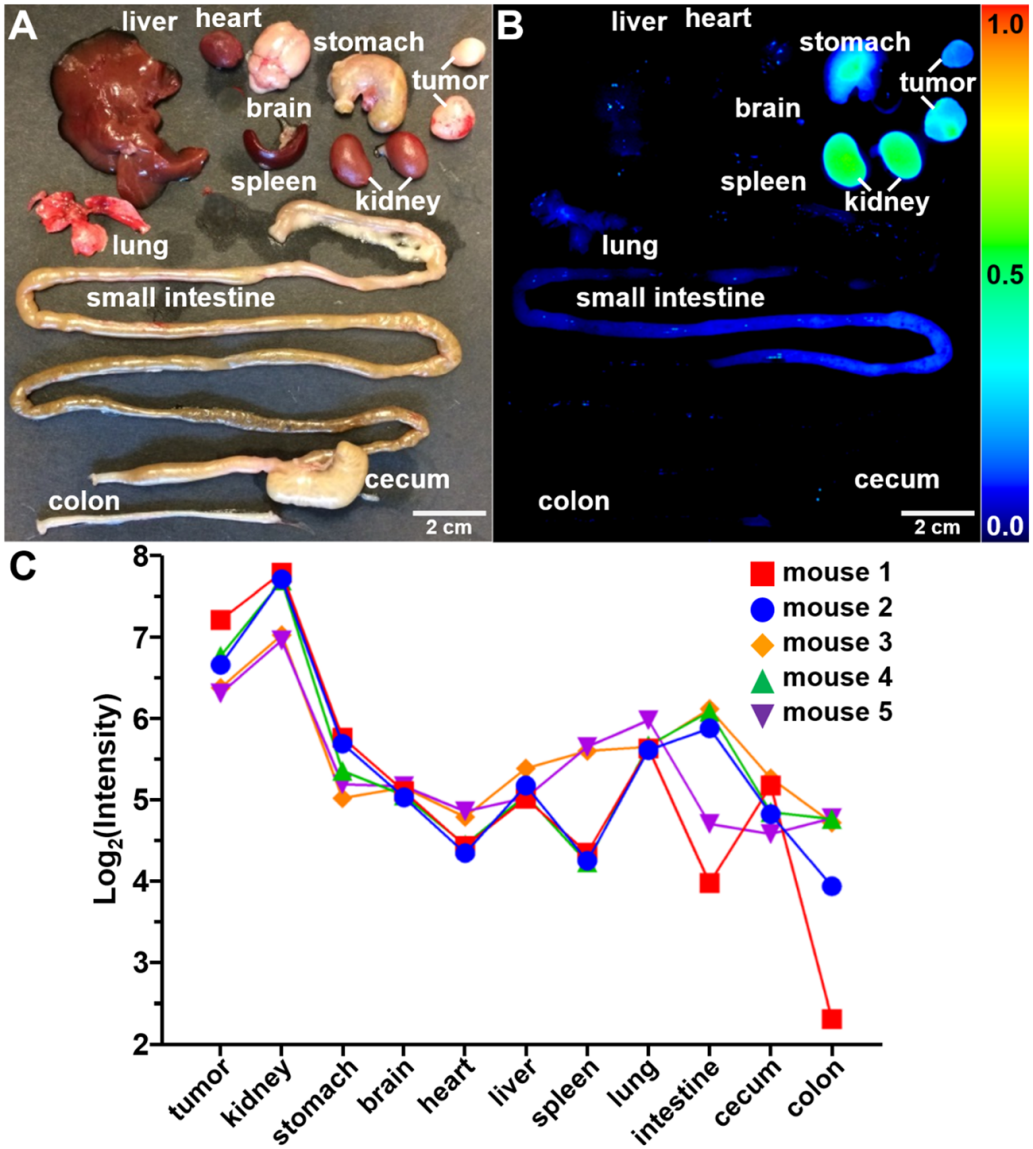
Peptide biodistribution. (**A**) White light image shows individual organs from tumor-bearing mouse euthanized 1 hour after injection of KSP*-IR800, including human breast cancer xenografts (tumors), liver, heart, stomach, brain, lung, spleen, kidney, small intestine, cecum, and colon. (**B**) Fluorescence image shows high peptide uptake in tumors by comparison with other organs. Strong signal from kidneys support renal clearance. (**C**) Quantified fluorescence intensities are shown for tumor and other organs from n = 5 mice. We fit a two-way ANOVA model to log-transformed data with terms for 11 tissues and 5 mice and tested each tissue mean against the mean for tumors. Kidney had 1.7 times more intense signals on average (*P* = 0.03), while all other tissues had at least 2-fold lower signals than tumor on average (*P* = 0.008 for lung was the largest *P*-value).

After completion of imaging, the mice were euthanized, and the tumors and internal organs were removed to evaluate KSP*-IR800 biodistribution, Fig. 5A,B. In n = 5 mice, we observed significantly greater fluorescence intensity from the tumor compared with that from other organs, Fig. 5C. High peptide uptake seen from the kidneys is consistent with renal clearance. We evaluated histology (H&E) and found no acute toxicity, Fig. S6A–J.

### Specific Peptide Binding to Human Xenograft Breast Tumors Expressing ErbB2

On immunofluorescence (IF), we observed strong binding of KSP*-IR800 to the surface (arrow) of BT474 (ErbB2+) cells in the human xenograft breast tumor sections, Fig. S7A, and minimal signal with PPS*-IR800, Fig. S7B. We quantified the fluorescence intensities, and found significantly higher mean value with KSP*-IR800 for BT474 versus MDA-MB-231 tumors, and that this difference was greater than that for PPS*-IR800, Fig. S7C. This result was confirmed qualitatively with immunohistochemistry (IHC) where intense reactivity is seen with anti-ErbB2 antibody to the surface (arrow) of BT474 cells, Fig. S7D. Representative histology (H&E) shows individual BT474 tumor cells (arrow), Fig. S7E. For MDA-MB-231 human xenograft breast tumor sections, we observed minimal signal on IF, Fig. S7F,G, and low reactivity with anti-ErbB2 on IHC, Fig. S7H. Representative histology (H&E) for the MDA-MB-231 tumor is shown, Fig. S7I. Similarly, we observed minimal signal with either peptide to normal on IF, Fig. S7J,K, and low reactivity on IHC, Fig. S5L. Representative histology (H&E) for normal is shown, Fig. S7M.

### Specific Peptide Binding to Human Breast Specimens Expressing ErbB2

**Figure 6:**
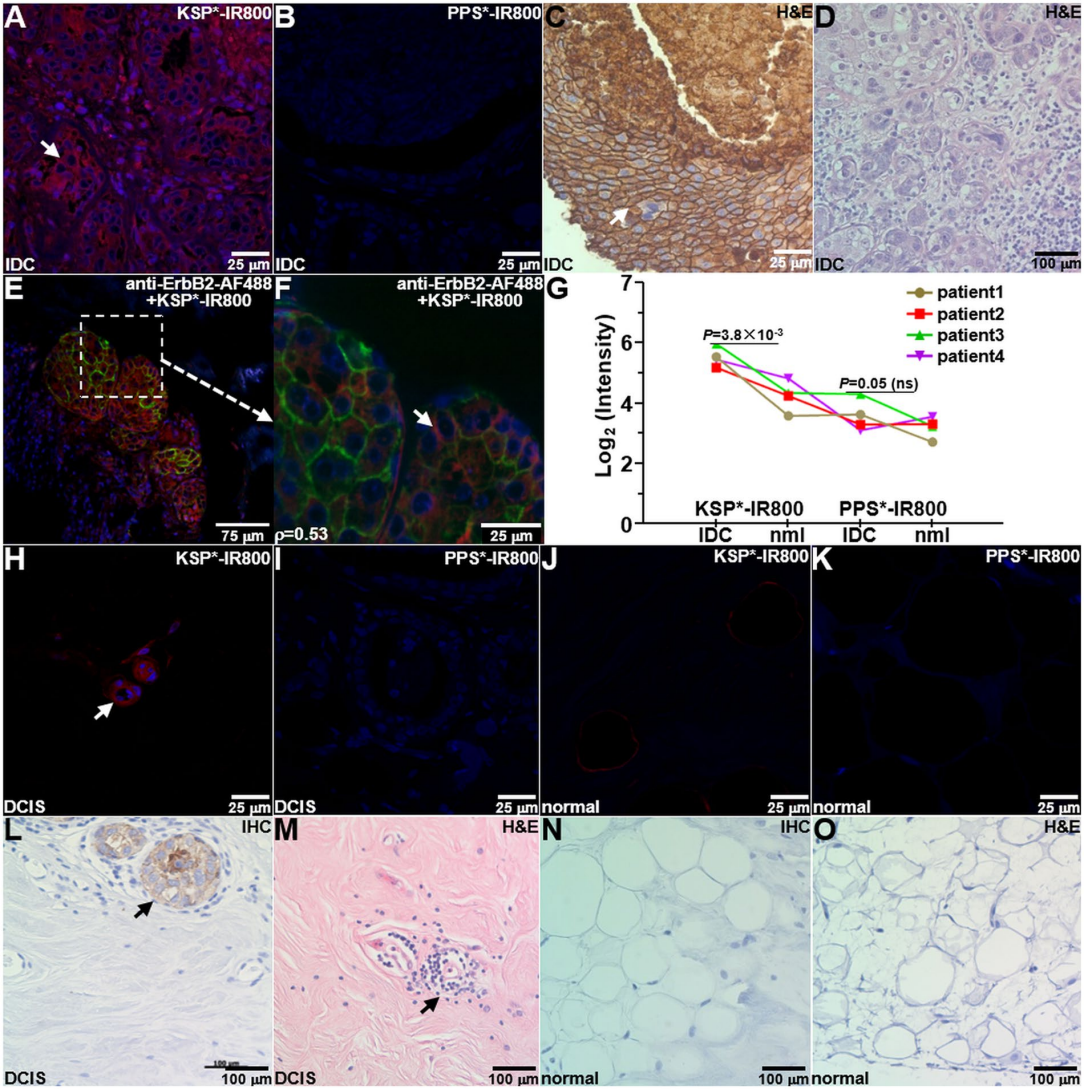
Specific peptide binding to human breast cancer *ex vivo*. (**A**) On immunofluorescence (IF), KSP*-IR800 shows strong staining to the surface (arrow) of invasive ductal carcinoma (IDC) cells from human specimens that express ErbB2, while minimal signal is seen with control (**B**) PPS*-IR800. (**C**) Immunohistochemistry (IHC) with anti-ErbB2 antibody confirms results. (**D**) Representative histology (H&E) for IDC. (**E**) Binding by KSP*-IR800 peptide (red) and anti-ErbB2-AF488 antibody (green) co-localizes on ErbB2 + IDC specimen with Pearson’s correlation coefficient of ρ = 0.53. (**F**) Magnified region from dashed box in (**E**) shows cell surface staining (arrow). (**G**) Quantitative comparison of KSP*-IR800 and PPS*-IR800 binding to human IDC (ErbB2+) with normal (ErbB2−) breast tissue. We fit an ANOVA model with terms for 4 conditions and 4 patients to log-transformed data and found a 2.44-fold greater signal for KSP*-IR800 in IDC than normal, but only a 1.30-fold increase for the same comparison with PPS*-IR800 peptide. The difference of differences was not significant, *P* = 0.09, which results from small number of human specimens. (**H**) On IF, we observed good staining of KSP*-IR800 to the surface (arrow) of non-invasive human ductal carcinoma *in situ* (DCIS) and (**I**) minimal signal with PPS*-IR800. On IF, we observed minimal staining to normal human breast tissue with either (**J**) KSP*-IR800 or (**K**) PPS*-IR800. (**L**) On IHC, we found strong reactivity to the surface (arrow) of DCIS cells. (**M**) Representative histology for DCIS. (**N**) Minimal reactivity was seen for normal human breast on IHC. (**O**) Representative histology for normal breast.

We evaluated peptide binding to sections of human breast specimens to assess potential for clinical translation. On IF, we observed strong binding of KSP*-IR800 to the surface (arrow) of invasive ductal carcinoma (IDC) cells, Fig. 6A, and minimal signal with PPS*-IR800, Fig. 6B. This result was confirmed qualitatively with immunohistochemistry (IHC) where intense reactivity is seen with anti-ErbB2 to the surface of IDC cells (arrow), Fig. 6C. Representative histology (H&E) is shown, Fig. 6D. We also observed strong co-localization of binding (arrow) between KSP*-IR800 peptide (red) and AF488-labeled anti-ErbB2 antibody (green) to ErbB2 + IDC specimens with Pearson’s correlation coefficient of ρ = 0.53, Fig. 6E,F. We quantified the fluorescence intensities, Fig. 6G. We also observed good binding of KSP*-IR800 to the surface (arrow) of non-invasive ductal carcinoma *in situ* (DCIS) cells on IF, Fig. 6H, and minimal signal with PPS*-IR800, Fig. 6I. We observed minimal signal with either peptide to normal breast on IF, Fig. 6J,K. This result was confirmed with IHC by reactivity seen with DCIS cells (arrow), Fig. 6L. Representative histology (H&E) for DCIS is shown, Fig. 6M. We observed low reactivity for anti-ErbB2 to normal breast on IHC, Fig. 6N. Representative histology (H&E) for normal is shown, Fig. 6O.

## Discussion

Here, we demonstrate a NIR-labeled ErbB2 peptide that can be used for *in vivo* imaging with either photoacoustics or fluorescence. Specific binding to ErbB2 was validated *in vitro* and *ex vivo* using standard lab assays. Following systemic administration, ErbB2 expression was visualized *in vivo* from human xenograft breast cancers implanted in nude mice. We used photoacoustic tomography to demonstrate rapid tumor uptake (∼1 hour), short circulatory half-life (<24 hours), and deep tissue imaging. We used a handheld dual-axes confocal fluorescence endomicroscope to collect optical sections in either the vertical or horizontal plane with sub-cellular resolution for real-time visualization with deep tissue penetration. The ErbB2 peptide cleared renally with minimal biodistribution outside of the tumor to suggest a safe toxicity profile. This peptide also showed strong binding *ex vivo* to IDC specimens. A scrambled peptide was used to rigorously control all studies. Our results demonstrate promise for future clinical use during breast cancer surgery to identify new lesions, guide intraoperative resection of cancer-positive lymph nodes, and provide rapid assessment of tumor margins.

Accurate assessment of ErbB2 in early breast cancer and metastases is clinically important because expression levels may correlate with tumor aggressiveness and disease-free survival, and may facilitate patient selection for ErbB2 specific therapies, dose adjustment, changes in treatment schedule, and monitoring of therapeutic response^18^. Several monoclonal antibodies and small molecule inhibitors have been used successfully to demonstrate the importance of ErbB2 in breast cancer pathogenesis^19,20^. IDC accounts for ~75% of all breast cancers^21^, while DCIS is the most common non-invasive form^22^. ErbB2 is expressed in up to 30% of all breast cancers and in up to 60% of non-invasive breast cancers. The sentinel lymph node (SLN) is an important target for *in vivo* imaging because it receives preferential tumor drainage from lymphatics, provides the primary conduit for cancer dissemination, and predicts the status of the other lymph nodes^23^. The handheld dual-axes confocal instrument may be used to perform SLN mapping to detect local metastases in breast conservation surgery, which is the preferred method for managing early stage disease^24^.

This peptide, labeled with Cy5.5, was shown previously to bind to ErbB2 extracellular domain 3 with fast kinetics (0.14 min^−1^) and high affinity (20 nM)^17^. We validated specific binding of this peptide labeled with IRDye800CW to ErbB2 using siRNA knockdown and competition with cells *in vitro* and with immunohistochemistry and immunofluorescence studies on tissue specimens using known monoclonal anti-ErbB2 antibodies. Also, we have previously used peptides labeled with fluorescein isothiocyanate (FITC) in human clinical studies in hollow organs^25,26^. We used IRDye800CW to image breast cancer, a solid organ, because of its greater imaging depth^27^. The absorption and emission peaks of the labeled and unlabeled peptides are similar, suggesting that chemical conjugation did not affect stability. Safety of IRDye800CW in Sprague-Dawley rats at intravenous doses up to 20 mg/kg has been previously published^28^, suggesting that this fluorophore may be non-toxic with human use. The monoclonal antibody trastuzumab has been labeled with IRDye800CW, and was found to achieve peak tumor uptake after a much longer duration of 12 days^29^.

Peptides offer a number of advantages for *in vivo* imaging to provide rapid feedback on ErbB2 receptor status during breast cancer surgery to improve the accuracy of SLN mapping^30^. Peptides clear rapidly, and result in reduced biodistribution to non-target tissues. While peptide binding affinities are not as high as that of antibodies for therapeutic purposes, they are adequate for diagnostic applications. Also, peptides are less immunogenic, which allows for repeated use^31^. Established peptide synthesis processes can be scaled up for mass manufacture with reproducible results at low cost using Good Manufacturing Practices (GMP) methods^32^. Moreover, peptides can be structurally altered to improve stability against proteolytic degradation by serum proteases^33^. They can be arranged in a multivalent configuration to improve binding affinity, sensitivity, and specificity for detecting early targets expressed at low levels^34^. Thess configuration can detect multiple targets concurrently to address tumor heterogeneity.

ErbB2 antibodies are being used clinically to detect breast cancer metastases *in vivo* with whole-body imaging methods, such as PET and SPECT^13,14^. Other targeting ligands are being evaluated in pre-clinical models. Antibody fragments, such as the Fab component of trastuzumab, have been radiolabeled with^111^In, and used to visualize human BT474 xenograft breast tumors as early as 24 hours post-injection^35^. By comparison, we were able to detect peak signal with an ErbB2 peptide at 1 hour post-injection. Affibody molecules are small, stable non-immunoglobulin scaffold-based proteins that have been radiolabeled with^111^In to show high uptake in human BT474 xenograft breast tumors at 1 hour post-injection with rapid clearance^36^. Also, a peptide radiolabeled with^111^In has shown good specificity for ErbB2 and has demonstrated good pharmacokinetic properties with SPECT imaging^37^. Antibodies^38^ and affibodies^39^ labeled with superparamagnetic iron oxide nanoparticles have been used to generate MR contrast, and have shown high uptake in human xenograft breast tumors. However, these whole-body imaging approaches are not well-suited for intra-operative use.

By comparison to whole body imaging methods, optical techniques do not involve ionizing radiation, are much safer, and less costly for clinical use. The dual-axes confocal endomicroscope uses a miniature scanner located in the distal end to provide a large FOV that is 4 times greater than that of other instruments of similar size. We used <2 mW of laser power incident on the tissue, a level that meets the FDA definition for non-significant risk^40^. This feature can accelerate clinical translation for sentinel lymph node mapping and early breast cancer staging. Because optical images are produced instantaneously, many lymph nodes can be examined rapidly to minimize the numbers required for resection. Time and cost associated with processing for either frozen sections or pathology can be reduced, and patients may need a shorter duration of anesthesia. Currently, use of non-specific radiolabeled colloid or blue dye for SLN mapping is limited by false negatives, and may take several hours to become visible^41^. Optical imaging may also reduce surgical complications associated with lymph node resection, such as lymphedema, which has been reported in up to 50% of patients^42^. In summary, we have developed a NIR-labeled peptide to detect *in vivo* ErbB2 expressed microscopically in human breast tumors using a miniature dual-axes confocal fluorescence endomicroscope to support future image-guided surgery.

## Materials and Methods

### Ethical Approval and Informed Consent

All experimental procedures were performed in accordance with relevant guidelines and regulations of the University of Michigan, and all animal studies were conducted with approval by the University Committee on the Use and Care of Animals (UCUCA). Animals were housed per guidelines of the Unit for Laboratory Animal Medicine (ULAM). All patient specimens were used with informed consent, and all experiments performed with human tissues were approved by the University of Michigan Institutional Review Board (IRB) under HUM00042180.

### Cells and Media

All human breast cancer cells were originally obtained from the ATCC (Manassas, VA). We used Roswell Park Memorial Institute (RPMI) medium for BT474, Dulbecco’s Modified Eagle Medium (DMEM) for MDA-MB-231, and McCoy’s medium for SKBR3 cells supplemented with 10% fetal bovine serum (FBS) and 1% penicillin/streptomycin. Penicillin/streptomycin was omitted for the siRNA knockdown studies. All cells were cultured at 37 °C with 5% CO_2_. The cells were passaged using 0.25% trypsin containing EDTA (Mediatech Inc). Western blot analysis was performed using a 1:1000 dilution of primary polyclonal rabbit anti-HER2 antibody (#2165, Cell Signaling Technology) per manufacturer instructions. Loading was controlled with a 1:500 dilution of monoclonal mouse anti-β-tubulin (#32–2600, Invitrogen). Cells were counted using a hemocytometer. Protein contents were quantified by bicinchoninic acid assay (BCA) assay.

### Peptide Specific for ErbB2 (HER2)

We synthesized the ErbB2 and control peptides using standard Fmoc mediated solid-phase chemical synthesis and labeled with either Cy5.5 or IRDye800 fluorophores via standard bio-conjugation techniques^43^. We used IRDye800CW-malemide (LiCor Biosciences) to label the peptide via a GGGSC linker at the C-terminus. The coupling yield was about 20–30% in aqueous solution without use of catalysis. IRDye800CW was used because of its high quantum yield, photostability, and lack of toxicity. The peptide absorption spectrum was measured using a UV-vis spectrophotometer in the 300–900 nm range (NanoDrop 2000, Thermo Scientific). Fluorescence emission from a 5 μM peptide solution diluted in PBS was collected with a fiber coupled spectrophotometer (Ocean Optics) using a diode pumped solid state laser (Technica Laser Inc) with excitation at λ_ex_ = 780 nm. The spectra were plotted using Origin 9.5 software (OriginLab Corp).

### Specific Peptide Binding to Cells Expressing ErbB2

~10^4^ human breast cancer cells were seeded on 1 mm thick cover glass, and grown to ~80% confluence in 12 well plates. The cells were gently washed with cold PBS and blocked with 2% BSA before incubation with peptides. 1 μM of IRDye800CW-labeled peptides mixed with 2% BSA was added to each well and incubated at 4 °C for 1 hour. After incubation for 5 min, the cells were washed with PBS 3X, fixed in ice cold 4% PFA for 10 min, and washed with PBS 1X. The cells were then embedded on microscope slides with 5 μL of ProLong Gold reagent containing DAPI (Invitrogen). SKBR3 cells were cultured in 6-well plates at 30–40% confluence in culture media supplemented with 10% fetal bovine serum without antibiotics. The cells were transfected with siRNA with a final concentration of 5 μM/L using oligofectamine (Thermo Scientific).

We examined ErbB2 knockdown in SKBR3 cells using ON-TARGETplus human siRNA (#L-003126–00–0005), ON-TARGETplus Non-targeting pool (#D-001810–10–05), and DharmaFECT transfection reagents (Thermo Scientific) per manufacturer instructions. We transfected SKBR3 cells with either siErbB2 (targeting siRNA) or siCL (non-targeting siRNA) for control at a final concentration of 5 µM/L using oligofectamine (Thermo Scientific).

Specific binding of KSP*-IR800 to SKBR3 cells was validated using competition with unlabeled KSP* peptide. SKBR3 cells were grown to ~70% confluence on coverslips in triplicate. Unlabeled KSP* at concentrations of 0, 100, 250, 500, and 1000 μM was incubated with the cells for 30 min at 4 °C. The cells were washed with PBS 3X, and further incubated with 0.5 μM of KSP*-IR800 for another 30 min at 4 °C. The cells were washed with PBS 3X and fixed with 4% PFA for 10 min. The cells were washed with PBS and then mounted with ProLong Gold reagent containing DAPI (Invitrogen). Confocal fluorescence images were collected and intensities from 6 independent images at each concentration were quantified using custom Matlab (Mathworks) software.

Binding co-localization was examined by first incubating the KSP*-IR800 peptide at 0.5 μM concentration with SKBR3 cells for about 1 hour at 4 °C. The cells were washed and fixed with 4% PFA for 5 min, and then incubated with primary anti-ErbB2 and secondary AF488-labeled antibody. We applied the peptide first due to its lower affinity, and used a low concentration (<1 μM) to minimize interference with antibody binding.

For imaging, we used an inverted confocal microscope (Olympus FV1200) with a 63X water immersion objective and λ_ex_ = 748 nm to excite IRDye800CW, λ_ex_ = 405 nm for DAPI, and λ_ex_ = 488 nm for Alexa Fluor 488. For antibody staining, the cells were pre-fixed with cold methanol for 10 min at −20 °C and blocked with 2% BSA for 30 min at room temperature (RT). Cells were incubated with a 1:450 dilution of anti-ErbB2 antibody (clone 111.6, Thermo Scientific) overnight at 4 °C. The cells were then washed with PBS 3X, and secondary goat anti-rabbit antibody labeled with Alexa-Fluor 488 (AF488) was added to the cells for 1 hour at RT. The cells were washed further with PBS and mounted onto cover glass. Fluorescence intensities were measured from 3 randomly positioned boxes with dimensions of 20 × 20 μm^2^, and were quantified using custom Matlab (Mathworks) software.

### *In Vivo* Imaging of Xenograft Breast Tumors

#### Mouse model

BT474 cells were diluted in growth factor reduced (GFR) Matrigel Matrix (Corning), and injected into one flank of female nude athymic mice (002019 Foxn1 <nu>, Jackson Laboratory) at 4–6 weeks of age with weight between 20–25 grams. A 0.18 mg 17-β-Estradiol pellet (#SE-121, Innovative Research of America) with 60 day release form was placed subcutaneously around the neck 1 day prior to tumor implantation to stimulate tumor growth. ∼1 × 10^7^ cells were implanted for each tumor, and 2–3 implantations were performed in each mouse. MDA-MB-231 tumors were generated similarly except without Matrigel Matrix injection and only one implantation was performed in each mouse. Tumor size was monitored by ultrasound and MRI weekly. For all *in vivo* animal experiments, anesthesia was induced and maintained with inhaled isoflurane mixed with oxygen at a concentration of 2–4% at a flow rate of ~0.5 L/min via a nose cone.

#### Ultrasound imaging

We collected ultrasound (US) images using a portable scanner (SonixTablet, Ultrasonix, Analogic Corp) designed for small animal imaging to measure tumor dimensions. The mice were placed on a heated stage to support body temperature. Tumors were covered with warm ultrasound gel (Aquasonic 100, Parker Laboratories) at 37 °C to facilitate imaging. We used a 40 MHz transducer in B-mode to measure the length, width, and height of the tumor to calculate the total volume. Each image had a 12 × 12 mm^2^ FOV with an in-plane pixel resolution of 50 × 50 μm^2^. Ellipsoid volumes (V) were estimated using the equation V = πabc/6, where a is the largest dimension in the sagittal plane, b is the value perpendicular to a, and c is the parameter orthogonal to both a and b in the transverse plane. Each measurement was performed 3 times and the mean value was used to estimate tumor size.

#### MR imaging

We collected MR images to verify tumor size. We used a 7 T horizontal bore small animal MRI system (SGRAD 205/120/HD/S, Agilent Technologies) with a volume-based transmit/receive quadrature radio frequency coil with an inner diameter of 3.5 cm^44^. Body temperature was maintained at 37 °C by blowing hot air into the magnet through a feedback control system. Transverse T_1_-weighted sections were acquired with a scout sequence in 3 orthogonal axes to identify tumor location. A 256 × 128 matrix was obtained in 5 min by conventional spin-echo multi-slice pulse sequence using repetition time (TR) = 8.5 ms, echo time (TE) = 2.6 ms, average = 2, in-plane FOV = 35 × 35 mm^2^, 25 mm slab thickness of 1 mm thick interleaved slices with no gap in between. Tumor volume was assessed with the freehand region of interest (ROI) function of NIH ImageJ software. Areas were measured on each MRI slice (1 mm thickness) and added together to reconstruct the 3D tumor volume.

#### Photoacoustic imaging

We used photoacoustic tomography (Nexus128, Endra Inc) to measure peptide uptake by the human xenograft breast tumors^45^. This system provides laser excitation with 7 ns pulses, 25 mJ/pulse, 20 MHz repetition rate, and tunable wavelength from 680 nm to 950 nm. The acoustic signal was collected with 128 unfocused (3 mm diameter) ultrasound transducers with 5 MHz center frequency assembled in a hemispherical bowl in a helical pattern. Water was added to the bowl to transmit the acoustic signal. We collected photoacoustic images with 120 views at 60 pulses/view. Each image covered a volume of 25 × 25 × 25 mm^3^ with a voxel size of 280 μm^3^, and each dataset required ~6–7 min for acquisition.

For imaging, the animals were placed inside a tray with the tumor positioned inside a water-filled dimple that couples the ultrasound signal. KSP*-IR800 was injected via tail vein at a concentration of 300 μM in a volume of 250 μL. Photoacoustic images were acquired at 0, 1, 2, 3, 6, 12, and 24 hours post-injection using λ_ex_ = 780 nm excitation. PPS*-IR800 was administered in the same animal 48 hours later for control after the target peptide cleared as defined by T/B ratio returning to ∼1. IRDye800 (fluorophore without peptide) at the same concentration was also administered for control.

After completion of imaging, 3D images were reconstructed using data acquired from all 128 transducers at each view using a back-projection algorithm to correct for variations in laser intensity from pulse-to-pulse and small changes in temperature that affect the velocity of acoustic waves in water. The reconstructed raw data was analyzed using Osirix 6.5.2 software (Pixmeo) to generate a maximum intensity projection (MIP) image. To calculate the T/B ratio, the mean photoacoustic signal was measured from the tumor (target) using a circular ROI function on 2D MIP images. For background, we used the same ROI as that for the tumor prior to injection of peptide. The adjacent annulus with same area was used measure the background.

#### Optical imaging

We identified the spatial extent and margins of the human xenograft breast tumors using a NIR whole body fluorescence imaging system (Pearl®, LI-COR Biosciences). Images with a FOV of 16.8 × 12 cm^2^ were collected with 85 μm resolution using λ_ex_ = 785 nm and λ_em_ = 820 nm. Anesthesia was provided during image acquisition using isofluorane via the SmartFlow Anesthesia Suite at a flow rate of 2%. Mouse body temperature was maintained at 37 °C. Images were analyzed with custom software (Image Studio, Li-Cor Biosciences). The region with an equal area adjacent to the tumor was used to measure background. Prism software (v6.02, GraphPad) was used to plot all data.

We evaluated ErbB2 expression with sub-cellular resolution by collecting optical sections *in vivo* using a handheld 5.5 mm diameter dual-axes confocal endomicroscope^9^. A solid-state diode laser (300 mW, CNI Laser Inc.) provides excitation at λ_ex_ = 785 nm. A parabolic mirror focuses the illumination and collection beams to overlap below the tissue surface with lateral and axial resolution of 2 and 5 μm, respectively. A compact, 3D monolithic scanner located in the distal tip provides large vertical displacements and wide angular deflections to produce images in the vertical (XZ) and horizontal (XY) planes with a FOV of 1000 × 430 and 1000 × 1000 μm^2^, respectively. NIR fluorescence is collected and passes through a long-pass edge filter (LP02–785RE-25, Semrock) that transmits from 790–1770 nm with >93% efficiency for detection with a photomultiplier tube (PMT, H7422PA-50, Hamamatsu) detector. We injected peptides via the tail vain, and dissected away the skin overlying the tumor for improved access. The distal tip of the endomicroscope was placed in contact with the tumor using a drop of saline to couple the light. Laser power <2 mW on the tissue was used to avoid photobleaching. NIR fluorescence images were collected at 5 frames per sec. 3D volumetric images were reconstructed from a series of images collected in the horizontal plane using Amira software (ver 5.4.3, FEI Corporation).

### Specific Peptide Binding to Human Xenograft Breast Tumors Expressing ErbB2

#### Immunofluorescence (IF)

Resected human xenograft breast tumors and normal breast tissues from n = 8 mice were formalin-fixed, paraffin embedded, and cut in 5 μm thick sections. IDC and normal human breast tissues were collected and prepared with the same protocol. The specimens were cut in 5 μm sections, and deparaffinization, rehydration and antigen unmasking was performed, as described previously^17^. Blocking was performed with DAKO protein blocking agent (X0909, DAKO) for 1 hour at RT. Sections were then incubated with 0.5 μM of either peptide in 2% BSA for 10 min at RT. The sections were washed with PBS 3X and mounted with Prolong Gold reagent containing DAPI (Invitrogen).

Formalin-fixed, paraffin-embedded (FFPE) human breast specimens known to express ErbB2 (n = 4 patients) were obtained from the archived tissue bank in the Department of Pathology at the University of Michigan. 5 μm sections were cut and rehydrated to water. Slides were counterstained with Harris Hematoxylin for 5 sec, dehydrated, and cover-slipped. Heat induced epitope retrieval was performed with FLEX TRS High pH Retrieval buffer (DAKO, 9.01) for 20 min. After peroxidase blocking, the antibody Her2/neu rabbit monoclonal (Cell Marque Corporation, SP3 cat#237R-16) was applied at a dilution of 1:150 at RT for 20 min. The EnVision + detection System HRP anti-rabbit (DAKO, #K4002) was used for detection. DAB chromogen was then applied for 10 min. Sections of human breast specimens were mounted onto glass slides (Superfrost Plus, Fischer Scientific). The tissues were deparaffinized, and antigen retrieval was performed, as discussed above. Histology and IHC were performed to evaluate for ErbB2 expression.

#### Immunohistochemistry (IHC)

Serial sections were prepared with 10 μm thickness and deparaffinized. Briefly, sections were incubated in xylene 3X for 3 min each, washed with 100% ethanol 2X for 2 min each, and washed with 95% ethanol 2X for 2 min each. Rehydration was performed by washing the sections 2x in dH_2_O for 5 min. Antigen unmasking was performed in boiled 10 mM citric acid buffer. After cooling at RT for 20–30 min, the sections were washed 3X in dH_2_O for 2 min each and in PBST for 5 min. Blocking was performed with DAKO protein blocking agent (X0909, DAKO) for 1 hour at RT. We used 1:250 dilution of monoclonal mouse anti-ErbB2 antibody (clone 111.6, Thermo Scientific). The sections were incubated overnight with primary antibody at 4 °C and washed 3X in PBS for 5 min. A 1:200 dilution of secondary antibody (goat anti-mouse) was added to each section and incubated for 30 min at RT. The secondary antibody solution was removed by washing 3X with PBS for 5 min. Premixed Elite Vectastain ABC reagent (Vector Labs) was added to each section and incubated for 30 min at RT. The sections were washed 3X in PBST for 5 min, and treated with 3,3′-diaminobenzidine substrate. The reaction was monitored for 1 min, and then quenched by immersing the slides in dH_2_O. Hematoxylin was added as a counterstain for ~20 sec, and the sections were dehydrated in increasing concentrations of ethyl alcohol (70%, 80%, 95% 2X, 100% 2X). Coverslips were attached using permount mounting medium (#SP15-100, Fisher) in xylene. Serial sections were processed for histology (H&E). Controls were prepared using secondary antibody, Elite Vectastain ABC reagent, Vector Labs and 3,3′-diaminobenzidine (without primary anti-ErbB2 antibody).

### Specific Peptide Binding to Human Breast Specimens Expressing ErbB2

Human breast specimens were embedded in paraffin and antigen unmasking was performed as described above. Blocking was performed with 2% BSA solution in PBS at RT for 1 hour. We used 1:250 dilution of monoclonal mouse anti-ErbB2 antibody (clone 111.6, Thermo Scientific). The sections were incubated at RT for 1 hour with primary antibody and then washed in PBS for 5 min. A 1:200 dilution of Alexa Fluor 488 (AF488) labeled secondary antibody (goat anti-mouse) was incubated with each section for 30 min at RT. The secondary antibody was removed by washing with PBS 3X for 5 min. Sections were then incubated with 0.5 μM KSP*-IR800 in 2% BSA for 10 min at RT. The sections were washed with PBS 3X and mounted with Prolong Gold reagent containing DAPI (Invitrogen). Confocal microscopy with 63X magnification was performed using excitation at λ_ex_ = 748, 488, and 405 nm to excite IRDye800CW, AF488, and DAPI, respectively. Fluorescence intensities were measured from 3 randomly positioned boxes with dimensions of 20 × 20 μm^2^.

### Data Availability

The datasets generated during and/or analyzed during the current study are available from the corresponding author upon request.

## Electronic supplementary material

Supplementary Information

Visualization1

Visualization2

Visualization3
