# Supplementary material for: In vivo near-infrared imaging of ErbB2 expressing breast tumors with dual-axes confocal endomicroscopy using a targeted peptide
Source: Sci Rep. 2017 Oct 31;7:14404. doi: 10.1038/s41598-017-13735-z (PMC5663926; doi:10.1038/s41598-017-13735-z)
Supplement: Supplementary file 1 — Supplementary Information [file 41598_2017_13735_MOESM1_ESM.pdf]

In vivo near-infrared imaging of ErbB2 expressing breast tumors with dual-axes confocal endomicroscopy using a targeted peptide

Zhenghong Gao,[1] Gaoming Li,[1] Xue Li,[1] Juan Zhou,[1] Xiyu Duan,[2] Jing Chen,[1] Bishnu P. Joshi,[1] Rork Kuick,[3] Basma Khoury,[1,4] Dafydd G. Thomas,[5] Tina Fields,[5] Michael S. Sabel[6] Henry D. Appelman,[5] Quan Zhou,[2] Haijun Li,[2] Ken Kozloff,[1,4] Thomas D. Wang[1,2,7]*

[1]Dept of Internal Medicine, University of Michigan, Ann Arbor, MI 48109, United States

[2]Dept of Biomedical Engineering, University of Michigan, Ann Arbor, MI 48109, United States

[3]Dept of Biostatistics, University of Michigan, Ann Arbor, MI 48109, United States

[4]Dept of Orthopaedic Surgery, University of Michigan, Ann Arbor, MI 48109, United States

[5]Dept of Pathology, University of Michigan, Ann Arbor, MI 48109, United States

[6]Dept of Surgery, University of Michigan, Ann Arbor, MI 48109, United States

[7]Dept of Mechanical Engineering, University of Michigan, Ann Arbor, MI 48109, United States

***Correspondence:**

Thomas D. Wang, M.D., Ph.D.
Professor of Medicine, Biomedical Engineering, and Mechanical Engineering
H. Marvin Pollard Collegiate Professor of Endoscopy Research
Division of Gastroenterology, University of Michigan
109 Zina Pitcher Pl. BSRB 1522
Ann Arbor, MI 48109-2200
Office: (734) 936-1228
Fax: (734) 647-7950
Email: thomaswa@umich.edu

Supplementary figures

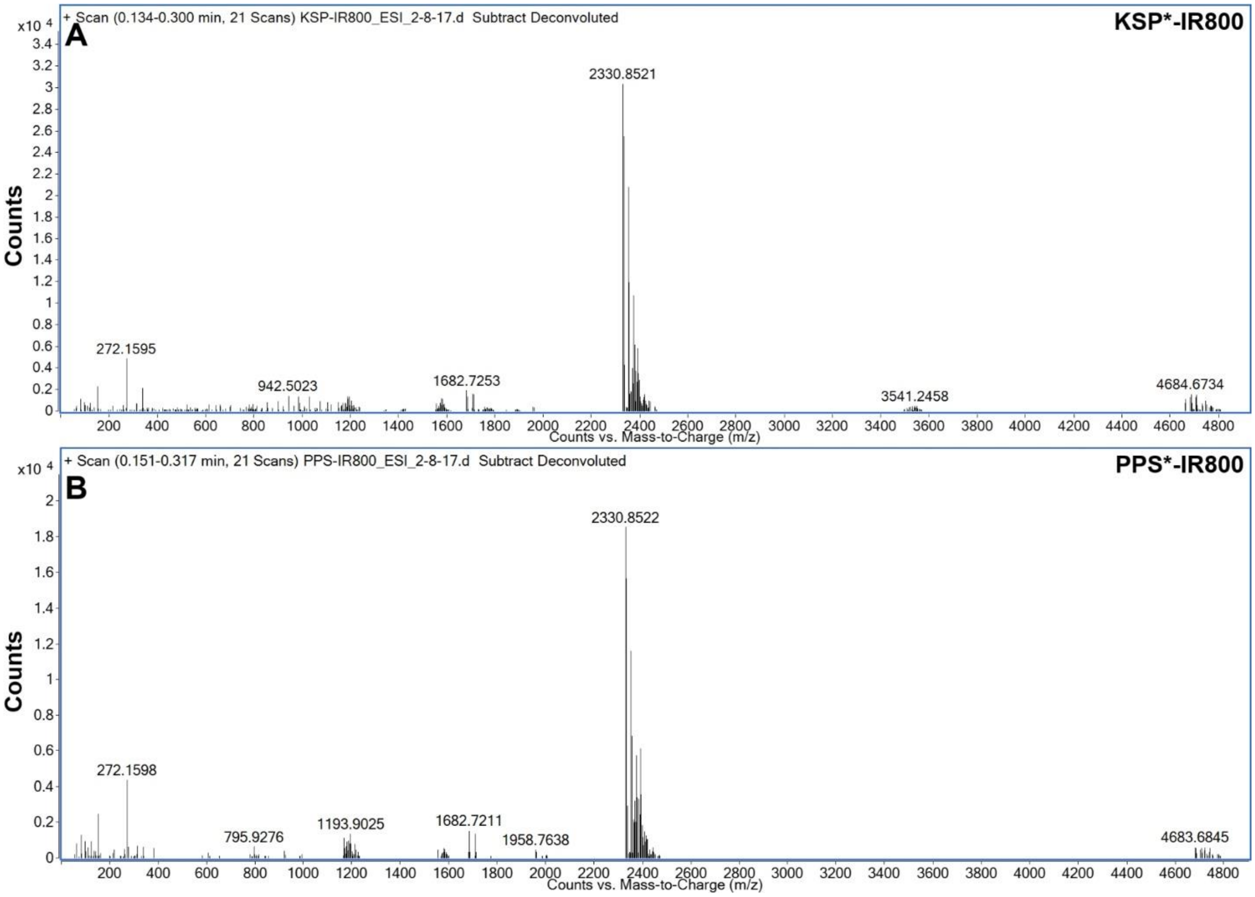

Figure S1 – Mass spec analysis. The experimental mass-to-charge (m/z) ratio of 2330.85 measured for **A**) KSP*-IR800 and **B**) PPS*-IR800 agrees with expected values.

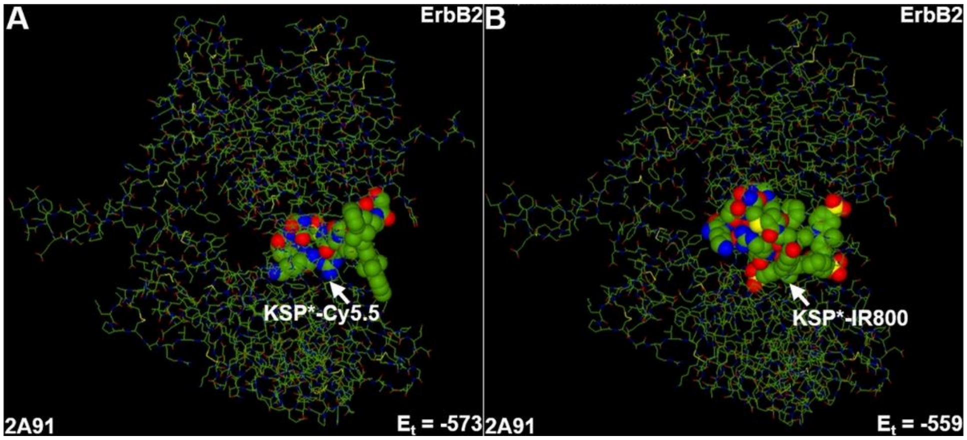

Figure S2 – Structural model. The total energy E_t calculated for binding of either **A**) KSP*-Cy5.5 or **B**) KSP*-IR800 to extra-cellular domain 3 of ErbB2 (2A91) is minimally affected by labeling with either Cy5.5 or IRDye800CW.

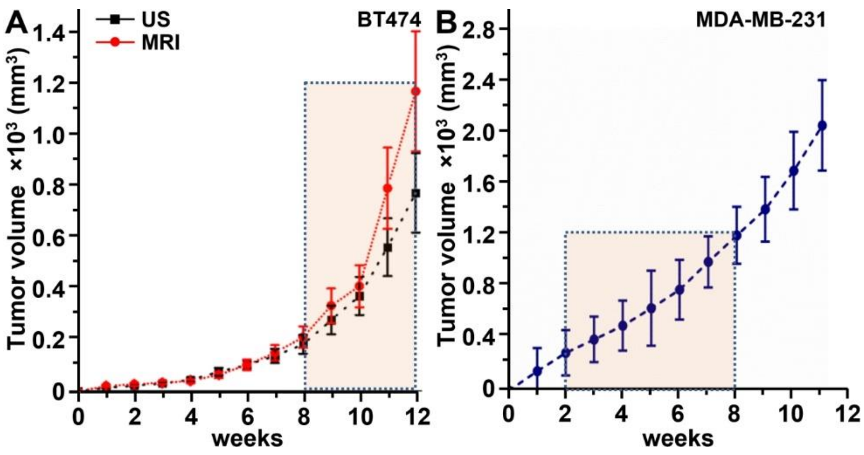

Figure S3 – Tumor dimensions. The mean (±SD) volume for **A**) BT474 human xenograft breast tumors (n = 8) in nude mice was measured with ultrasound (US) and T_1-weighted MRI over 12 weeks. The size of **B**) MDA-MB-231 tumors was measured with calipers. Dashed boxes show period of time that multi-modal imaging was performed using the ErbB2 peptide.

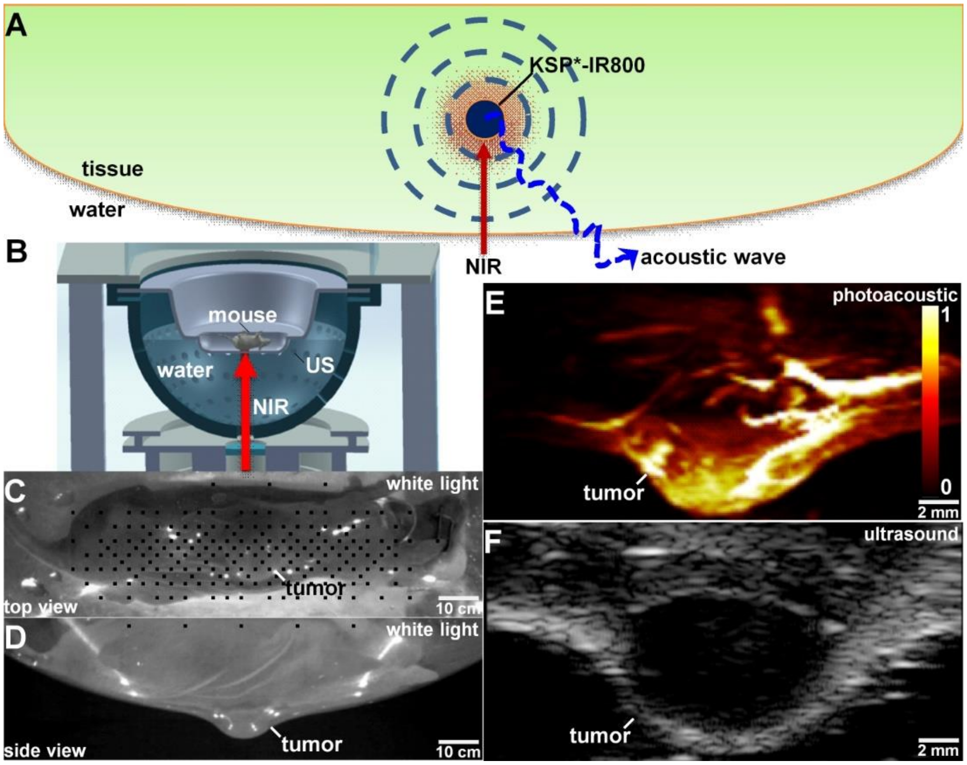

Figure S4 – Photoacoustic imaging. **A**) Acoustic wave is generated from NIR light absorbed by fluorophore. **B**) Tumor-bearing mice are placed face up in a water-filled hemispherical bowl that contains 128 unfocused ultrasound transducers arranged in a helical pattern. Photograph shows **C**) top and **D**) side-view of mouse with tumor placed in a water-filled dimple. **E**) Photoacoustic and corresponding **F**) ultrasound image from same tumor collected in vivo shows vertical cross-section of tumor.

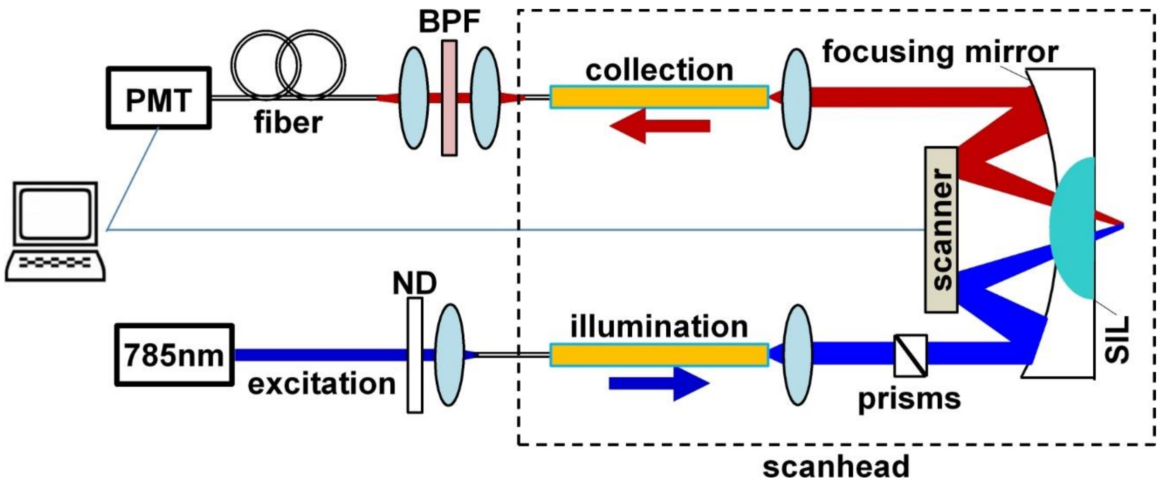

Figure S5 – Schematic. In the 5.5 mm diameter handheld dual-axes confocal endomicroscope, excitation is provided at λ_{ex} = 785 nm. Separate illumination and collection beams travel along different paths at an angle into the tissue. The region of overlap defines the focus. This off-axis geometry minimizes the effects of tissue scattering, and enhances the dynamic range of detection so that images can be collected in vertical as well as horizontal cross-sections. A miniature scanner produces wide angular deflections and large vertical displacements to generate large fields-of-view (FOV) in either the vertical or horizontal planes.

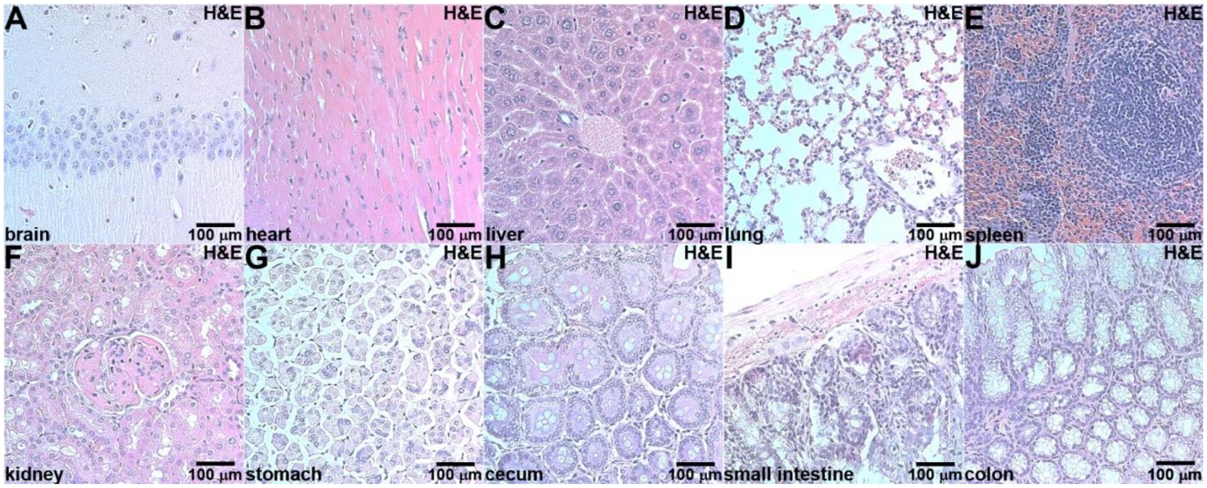

Figure S6 – No acute peptide toxicity. Nude mice bearing BT474 human xenograft breast tumors were sacrificed at 1 hour post injection of KSP*-IR800. No signs of acute peptide toxicity were seen on histology (H&E) in **A**) brain, **B**) heart, **C**) liver, **D**) lung, **E**) spleen, **F**) kidney, **G**) stomach, **H**) cecum, **I**) small intestine, and **J**) colon.

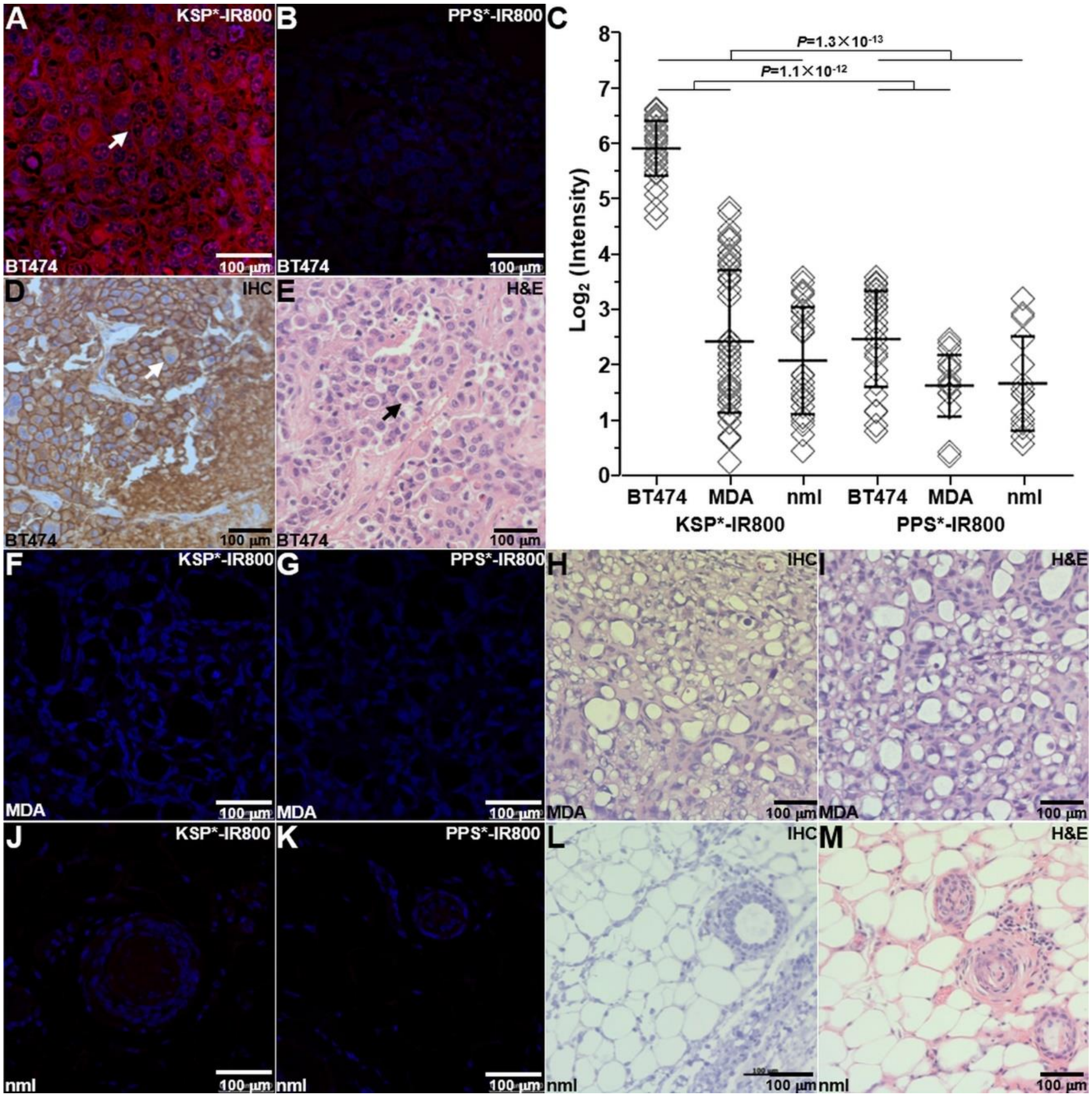

Figure S7 – Xenograft breast tumor sections. On immunofluorescence (IF), we observed strong staining by **A**) KSP*-IR800 but not **B**) PPS*-IR800 to the surface (arrow) of BT474 cells. **C**) Quantitative comparison of binding by KSP*-IR800 and PPS*-IR800 to BT474 and MDA-MB-231 (ErbB2-) tumors and normal breast is shown. For KSP*-IR800, we found a significantly larger difference for BT474 versus MDA-MB-231 than the same difference for PPS*-IR800, with P=1.1×10^{-13} with the fold increase being 7.4 times larger (13.4 versus 1.8). Similarly, the difference for BT474 tumor versus normal breast was 8.2 times larger for KSP*-IR800 than for PPS*-IR800, P=1.3×10^{-12}. The difference of differences was determined using an ANOVA with terms for 6 groups on log-transformed data. **D**) Intense reactivity to the surface (arrow) of BT474 cells was seen on immunohistochemistry (IHC) using anti-ErbB2. **E**) Representative histology (H&E) of BT484 tumor is shown. On IF, minimal fluorescence intensity is seen in MDA-MB-231 tumors with either **F**) KSP*-IR800 or **G**) PPS*-IR800. **H**) Low reactivity is seen with MDA-MB-231 tumors on IHC. **I**) Representative histology (H&E) is shown for MDA-MB-231 tumor. On IF, minimal fluorescence intensity is seen for normal breast with either **J**) KSP*-IR800 or **K**) PPS*-IR800. **L**) No reactivity is seen for normal breast on IHC. **I**) Representative histology (H&E) of normal breast is shown.

Video legends

Visualization1 – Real-time optical sections of ErbB2 expression in breast tumor collected in vivo after 1 hour administrated KSP*-IR800 with handheld dual-axes confocal endomicroscope in the vertical plane from the human breast cancer xenograft.

Visualization2 – Real-time optical sections of ErbB2 expression collected in vivo after 1 hour administrated KSP*-IR800 with handheld dual-axes confocal endomicroscope in the horizontal plane from the human breast cancer xenograft.

Visualization3 – Reconstructed 3-dimensional volumetric image.

Original Western blots for Fig. 2F:

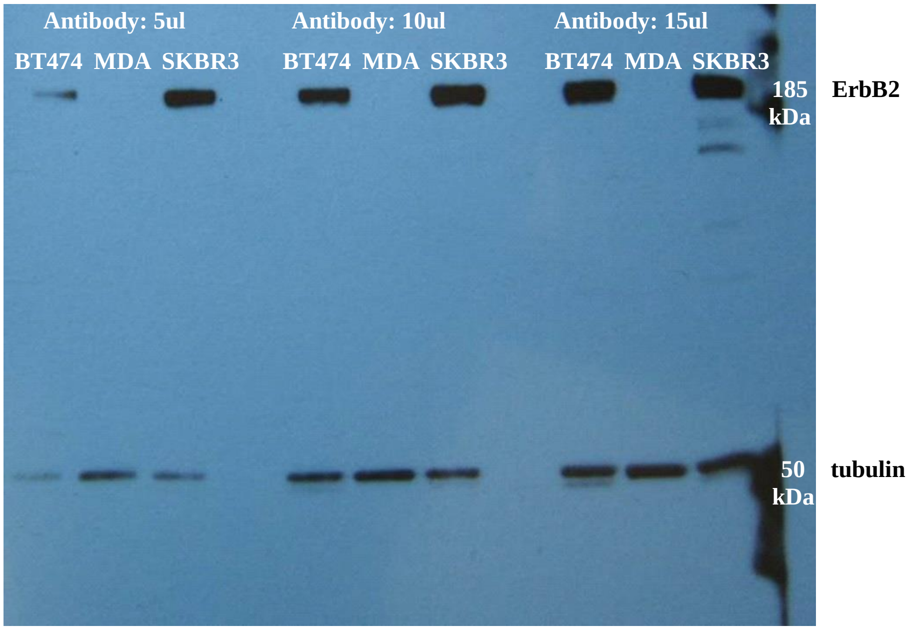

Original Western blots for Fig. 2G:

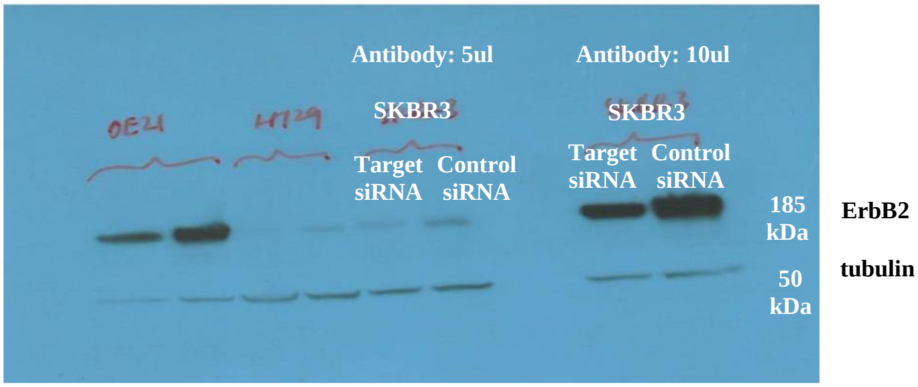

Note: for this experiment, the part of the gel containing no interested blots was cut off and removed, and only the parts with ErbB2 and tubulin blots were put together and exposed.
